# Stroke, multimorbidity and polypharmacy in a nationally representative sample of 1,424,378 patients in Scotland: implications for treatment burden

**DOI:** 10.1186/s12916-014-0151-0

**Published:** 2014-10-03

**Authors:** Katie I Gallacher, G David Batty, Gary McLean, Stewart W Mercer, Bruce Guthrie, Carl R May, Peter Langhorne, Frances S Mair

**Affiliations:** Institute of Health and Wellbeing, University of Glasgow, Glasgow, G12 9LX Scotland; Department of Epidemiology and Public Health, University College London, London, WC1E 6BT England; University of Edinburgh, Centre for Cognitive Ageing and Cognitive Epidemiology, Edinburgh, EH8 9JZ Scotland; University of Dundee, School of Medicine, Dundee, DD2 4BF Scotland; University of Southampton, Faculty of Health Sciences, Southampton, SO17 1BJ England; Institute of Cardiovascular and Medical Sciences, University of Glasgow, Glasgow, G4 0SF Scotland

## Abstract

**Background:**

The prevalence of multimorbidity (the presence of two or more long-term conditions) is rising internationally. Multimorbidity affects patients by increasing their burden of symptoms, but is also likely to increase the self-care demands, or treatment burden, that they experience. Treatment burden refers to the effort expended in operationalising treatments, navigating healthcare systems and managing relations with healthcare providers. This is an important problem for people with chronic illness such as stroke. Polypharmacy is an important marker of both multimorbidity and burden of treatment. In this study, we examined the prevalence of multimorbidity and polypharmacy in a large, nationally representative population of primary care patients with and without stroke, adjusting for age, sex and deprivation.

**Methods:**

A cross-sectional study of 1,424,378 participants aged 18 years and over, from 314 primary care practices in Scotland that were known to be demographically representative of the Scottish adult population. Data included information on the presence of stroke and another 39 long-term conditions, plus prescriptions for regular medications.

**Results:**

In total, 35,690 people (2.5%) had a diagnosis of stroke. Of the 39 comorbidities examined, 35 were significantly more common in people with stroke. Of the people with a stroke, the proportion that had one or more additional morbidities present (94.2%) was almost twice that in the control group (48%) (odds ratio (OR) adjusted for age, sex and socioeconomic deprivation 5.18; 95% confidence interval (CI) 4.95 to 5.43). In the stroke group, 12.6% had a record of 11 or more repeat prescriptions compared with only 1.5% of the control group (OR adjusted for age, sex, deprivation and morbidity count 15.84; 95% CI 14.86 to 16.88). Limitations include the use of data collected for clinical rather than research purposes, a lack of consensus in the literature on the definition of certain long-term conditions, and the absence of statistical weighting in the measurement of multimorbidity, although the latter was deemed suitable for descriptive analyses.

**Conclusions:**

Multimorbidity and polypharmacy were strikingly more common in those with a diagnosis of stroke compared with those without. This has important implications for clinical guidelines and the design of health services.

**Electronic supplementary material:**

The online version of this article (doi:10.1186/s12916-014-0151-0) contains supplementary material, which is available to authorized users.

## Background

Multimorbidity, defined as the presence of two or more long-term conditions, is becoming a global challenge for policy-makers, clinicians, and patients [1-3]. Treatment advances and increasing sub-specialisation of health services have improved functional outcomes for those with long-term conditions, but such changes have resulted in an increasing burden of treatment demands on patients, particularly those with multimorbidity [3,4]. Treatment burden is defined as the workload of healthcare for patients and the impact of this on their wellbeing [5]. It includes information gathering, attending multiple appointments, taking medications, enacting self-care, and, in countries that lack a health service that is free at the point of care, organising finances to pay for treatments [5-8]. There is a risk that patients become overburdened by their treatments, which can mean failure to adhere to management plans, thus resulting in ineffective treatment and wasted resources [3,9-11].

One aspect of treatment burden described above is polypharmacy, which can contribute to other treatment burdens such as adverse drug events [12,13]. Polypharmacy is most commonly defined as the use of multiple (usually five or ten) prescribed medications [14-16]. Although there is no strong evidence to support the use of any particular threshold, the risk of drug-related problems seems to increase with each additional medication prescribed [17,18]. There is a known association between number of morbidities and polypharmacy [19-21], with a study using routine Scottish health records finding that of those with two clinical conditions, 20.8% were receiving four to nine medications, and 1.1% were receiving ten or more medications; for patients with six or more comorbidities, these values were 47.7% and 41.7%, respectively [19]. A systematic literature review investigating the relationship between the number of chronic conditions and healthcare utilisation outcomes found that about 60% of elderly respondents with zero or one condition reported taking prescription medications. This percentage went up to more than 90% for those with two or three conditions, and approached 100% for those with more than five conditions [20], supporting the premise that those with higher numbers of conditions to manage are more likely to experience higher levels of treatment burden [3]. Other aspects of treatment burden such as healthcare utilisation have also been shown to be associated with multimorbidity [20,22].

Stroke is a condition that can have a considerable impact on an individual’s life. A recent systematic review of the qualitative literature revealed that people who have had a stroke experience four main areas of treatment burden: making sense of stroke management and planning care, interacting with others, enacting management strategies, and reflecting on management [23]. Poor communication between patients and professionals was a common experience, exacerbated by fragmentation of health services and poor communication between healthcare providers themselves, aspects of stroke care likely to be exacerbated by multimorbidity [24-26]. Surprisingly, there has been limited exploration of multimorbidity or polypharmacy in people with stroke, the field being characterised by small-scale studies and a small number of conditions under examination [19,27-36]. Those studies that have examined stroke in relation to other long-term conditions have suggested that stroke is one of the diseases most significantly associated with polypharmacy [19,33], but there is a lack of large-scale studies examining a broad range of medications and comorbidities.

In the current study, using a large, nationally representative cross-sectional primary care dataset, we examined the prevalence of multimorbidity and polypharmacy in people with and without stroke.

## Methods

### Study design and participants

This was a cross-sectional study based on a nationally representative dataset managed by the Primary Care Clinical Informatics Unit at the University of Aberdeen in Scotland. This fully anonymised dataset contains clinical data on all people that were alive and permanently registered with 314 primary care practices in Scotland on 31 March 2007. Comprising approximately one-third of the Scottish adult population, this sample has been shown to be representative of this population [37]. In the UK, registration with a medical practice is required for an individual to access National Health Service (NHS) healthcare in the community. It is estimated that over 98% of the population are registered with a medical practice [38], which systematically records information on each patient in an electronic medical record, for the purposes of registration and subsequent everyday medical care. We examined data extracted from medical records and collated for a previous study of multimorbidity that had examined the presence of forty conditions [1]. The NHS National Research Ethics Service approved the use of these data for research purposes. Patient consent was not deemed necessary due to full anonymisation of the data.

### Data collected and disease definition

The data examined consisted of the following variables: sex, age, socioeconomic deprivation (measured from patients’ postcodes using the Carstairs score [39]), counts of regularly prescribed medications and the presence of 40 long-term conditions, including stroke.

There is no ‘gold standard’ method for the measurement of multimorbidity, therefore the forty long-term conditions included had been chosen and defined based on a recent systematic review [40] and expert consensus [1]. Existing definitions for each long-term condition were used if possible, mainly those used in the Quality and Outcomes Framework (QOF) or by NHS Scotland [1,41,42]. If no standard definition was available, or there was concern about under-recording, then conditions were defined by the clinical members of the research team. For example, depression was defined as the presence of a QOF Read Code in the past year or receipt of four or more prescriptions for antidepressant drugs (excluding low-dose tricyclics, which are usually used for chronic pain) in the past year [1]. The definitions of all morbidities examined are given in supplementary material (see Additional file 1). Comorbidity was measured using a count of long-term conditions [43], with morbidities being noted as either mental health or physical morbidities. The original analysis measured the presence of a combined group of stroke or transient ischaemic attack (TIA), but for the purposes of this analysis, the presence of stroke alone was defined using the QOF Business Rules code set [41], and TIA was ignored.

As there are no standard definitions of regularly prescribed treatments or measure of polypharmacy, we utilised a count of current regular prescriptions, including tablets, inhalers, stoma care and topical therapies [17,18]. Regular (‘repeat’) prescriptions are clearly distinguished in UK general practice electronic medical records from one-off (‘acute’) prescriptions such as those for most antibiotics. For the purposes of this analysis, any regular prescription that was still active (that is, available for issue on request) on the date of extraction and that had been prescribed in the past 84 days was counted as current. This time frame was selected as this was the maximum length of a repeat prescription in Scotland at the time of data collection.

### Statistical analysis

Analyses were predicated on a comparison of the characteristics of people with stroke (cases) and those without stroke (controls). First, the numbers of morbidities and prescribed medications in stroke cases and controls were calculated, and proportions within each group computed. Second, logistic regression, which produces ORs, was used to summarise the relationship between stroke and the presence of comorbidities and prescribed medications. ORs were initially unadjusted – for the purposes of comparison – then adjusted for the key confounding factors of age, sex and socioeconomic deprivation. Age and deprivation were used as continuous variables. Deprivation was measured using the Carstairs score, which is widely used in health research. The Carstairs score is based on four census indicators: low social class, lack of car ownership, overcrowding and male unemployment. The scores have been described as a measure that reflects access to ‘those goods and services, resources and amenities and of a physical environment which are customary in society’ [39]. The scores therefore cannot be described as a measure of the extent of an individual’s material wellbeing, but are rather a summary measure applied to populations contained within small geographic localities. Further adjustment for number of morbidities was made when polypharmacy was the characteristic of interest. Associations between numbers of morbidities and prescriptions were assessed using Spearman correlation coefficients. For the purposes of this analysis, a *P* < 0.05 was deemed statistically significant. All analyses were carried out using IBM Statistical Package for the Social Sciences (SPSS) Statistics software (V21).

## Results

The analyses were based on 1,424,378 individuals (724,949 women) aged 18 years and over who were registered with a general practitioner. In total, 35,690 people (2.5%) had a diagnosis of stroke. As anticipated, the mean age of people in the stroke group (72.68 ± 12.21) was higher than that of the controls 47.36 ± 17.93). For the demographic characteristics for each group, see Additional file 2.

### Co*morbidities*

Table 1 shows the number and percent of total morbidities, physical morbidities and mental health morbidities in the stroke and control groups, along with ORs for stroke in relation to these variables. Multimorbidity was common in stroke: of the study members with stroke, the percentage that had one or more additional morbidities present (94.2%) was almost twice that in the control group (48%) (OR adjusted for age, sex and deprivation 5.18; 95% CI 4.95 to 5.43). Disaggregating the data into type of morbidity revealed that physical morbidity was markedly more common in people with stroke (adjusted OR 4.50; 95% CI 4.31 to 4.68), and mental health morbidity was also more common but the relationship was less strong (adjusted OR 2.10; 95% CI 2.05 to 2.15). In terms of assessing whether these differences exist across different age groups, a sub-analysis for age groups 35–44 years and 75+ years was performed (see Additional file 3). This indicated that differences were larger for the younger age group, and increased with the number of conditions (a similar picture was found for number of repeat prescriptions). However, the skewed distribution of stroke prevalence towards the oldest age groups make any assessment of differences by age problematic, owing to the small sample sizes in the youngest age groups.

**Table 1:** **Stroke status and number of morbidities (N = 1,424,378)**

	**Stroke N (%)**	**No stroke (%)**	**Unadjusted OR (95% ** **CI)** ^**a**^	**Age, gender and deprivation adjusted OR (95% ** **CI)** ^**a**^
	**35690 (100)**	**1388688 (100)**		
Total number of morbidities^b^				
None	2053 (5.8)	721430 (52.0)	1	1
One-three	17750 (49.7)	551295 (39.7)	11.31 (10.81 to 11.85)	4.35 (4.15 to 4.56)
Four-six	12300 (34.5)	100500 (7.2)	43.01 (41.03 to 45.09)	8.59 (8.17 to 9.04)
Seven or more	3587 (10.1)	15463 (1.1)	81.52 (77.04 to 86.26)	12.81 (12.05 to 13.61)
Number of physical morbidities^b^				
None	2769 (7.8)	800202 (57.6)	1	1
One-three	20716 (58.0)	510846 (36.8)	11.72 (11.26 to 12.20)	4.03 (3.86 to 4.20)
Four-six	10414 (29.2)	70709 (5.1)	42.56 (40.79 to 44.41)	7.32 (6.99 to 7.67)
Seven or more	1791 (5.0)	6931 (0.5)	74.68 (70.05 to 79.61)	10.33 (9.64 to 11.05)
Number of mental morbidities				
None	21961 (61.5)	1163095 (83.8)	1	1
One-three	13533 (37.9)	223739 (16.1)	3.20 (3.13 to 3.27)	2.08 (2.04 to 2.13)
Four or more	196 (0.5)	1854 (0.1)	5.60 (4.83 to 6.49)	3.56 (3.03 to 4.20)

^a^all p < 0.001.

^b^excluding stroke.

The ten most frequent comorbidities present in people with a diagnosis of stroke were: hypertension (60.9%), coronary heart disease (29.5%), painful condition (21.9%), depression (20.7%), diabetes (18.8%), chronic kidney disease (14.3%), constipation (13.8%), atrial fibrillation (13.0%), thyroid disorders (11.9 %), and chronic obstructive pulmonary disease (11.9%). Prevalences of all morbidities are shown in supplementary material (see Additional files 4 and 5).

**Figure 1:**
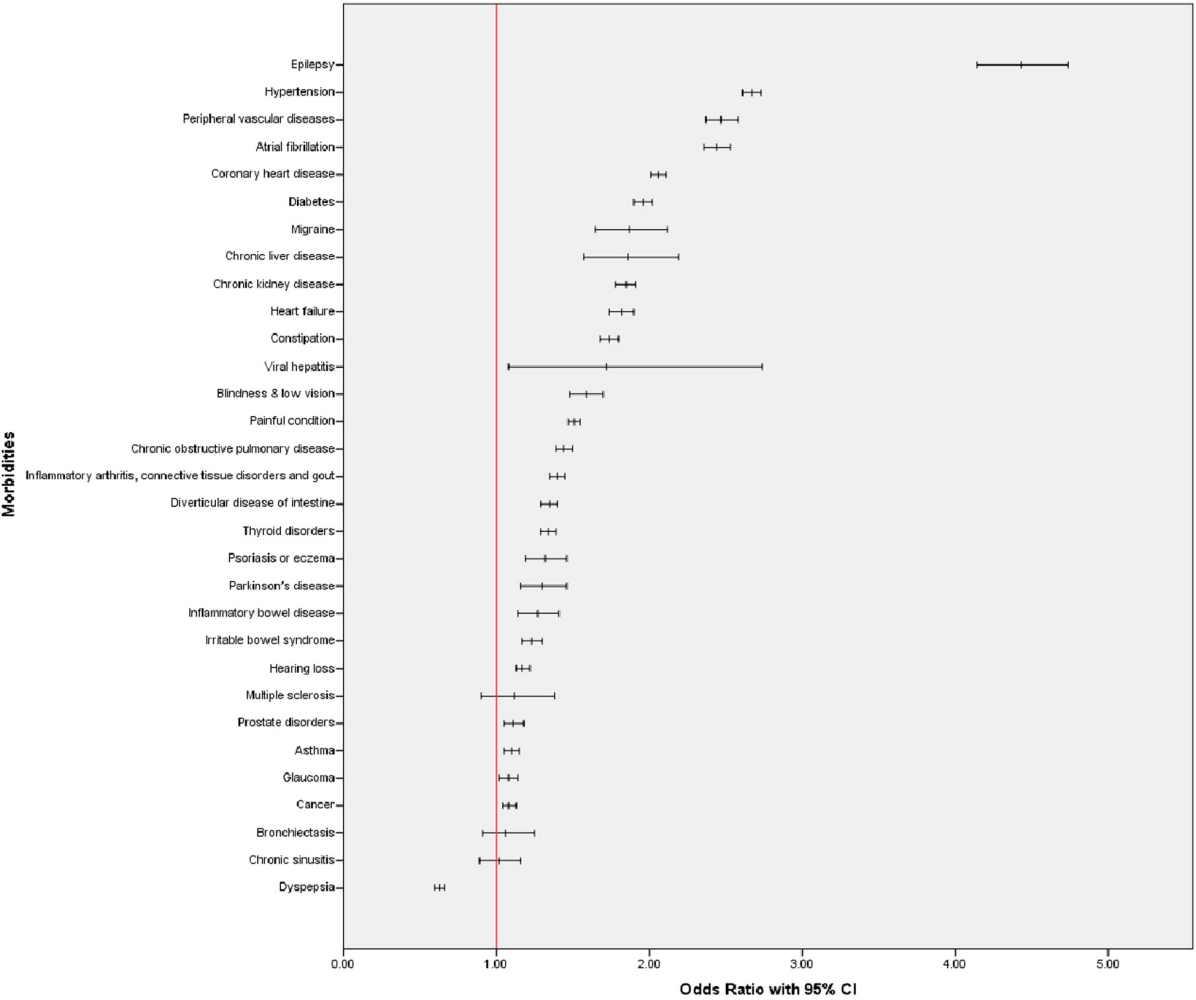
**Odds ratios (with 95%** **Cl) for physical morbidities in relation to stroke status (adjusted for age, sex, and deprivation).** The stroke group comprised 35,690 people, and the stroke-free group comprised 1,388,688 people.

**Figure 2:**
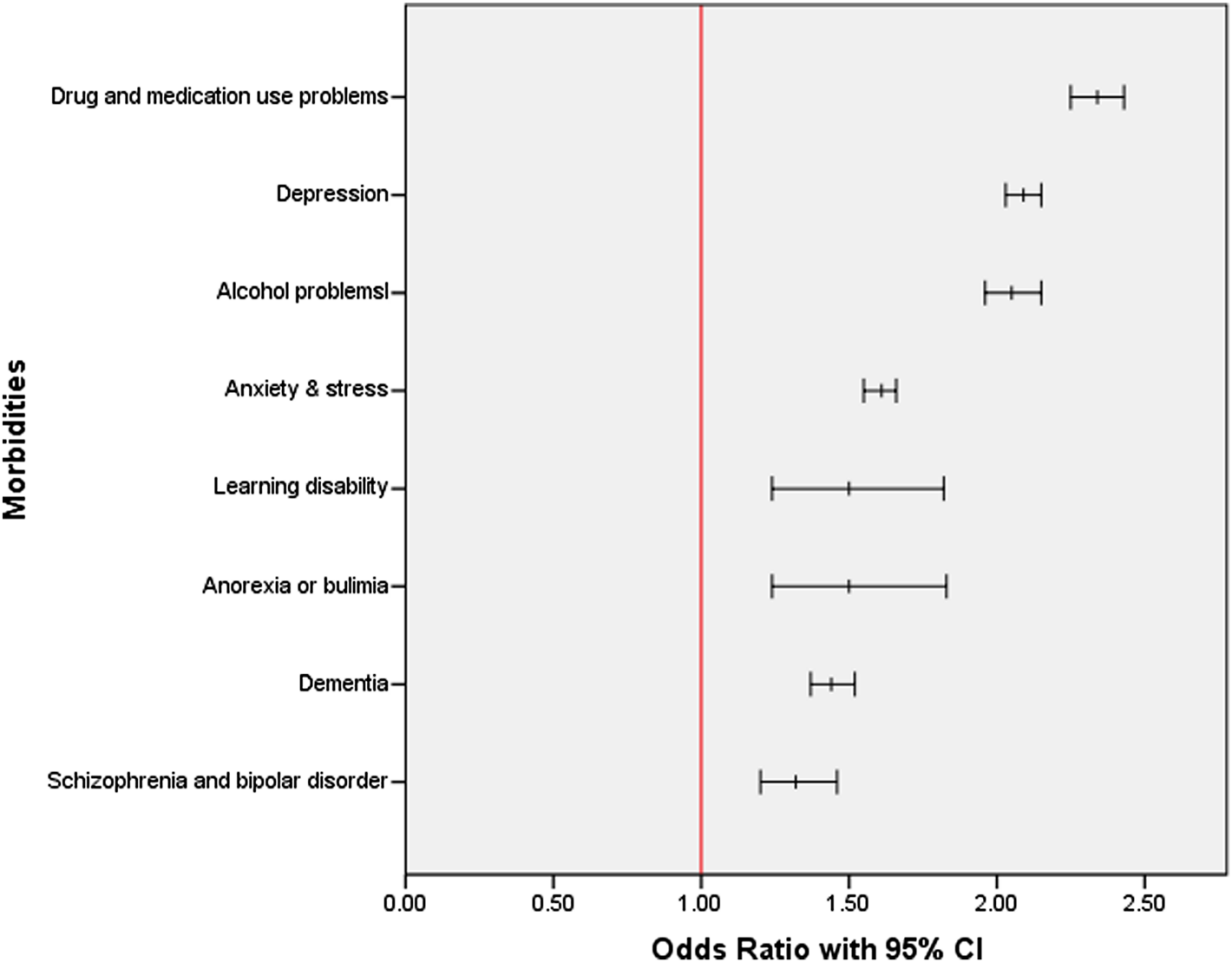
**Odds ratios (with 95%** **Cl) for mental health morbidities in relation to stroke status (adjusted for age, sex, and deprivation).** The stroke group comprised 35,690 people, and the stroke-free group comprised 1,388,688 people. Note: Drug related problems is any Read code which records psychoactive substance abuse which includes both drug misuse and prescription drug problems of multiple sources.

Figure 1 displays the ORs (adjusted for age, sex and deprivation) for stroke in relation to the thrity one physical morbidities examined. The supplementary material (see Additional file 4) elaborates on this by showing both the unadjusted and adjusted ORs along with the crude prevalence of all physical morbidities in the stroke and control groups. In all, twenty eight of the thirty one physical morbidities examined were significantly more common in the stroke group, this was twenty seven after adjustment for potential confounding factors. For instance, epilepsy (adjusted OR 4.43; 95% CI 4.14 to 4.74), hypertension (adjusted OR 2.67; 95% CI 2.61 to 2.73), peripheral vascular disease (adjusted OR 2.47; 95% CI 2.37 to 2.58), AF (adjusted OR 2.44; 95% CI 2.36 to 2.53) and CHD (adjusted OR 2.06; 95% CI 2.01 to 2.11) were all more common in people experiencing a cerebrovascular disease event. By contrast, dyspepsia was markedly less common in the stroke group (adjusted OR 0.63; 95% CI 0.60 to 0.66). Figure 2 shows the ORs (adjusted for age, sex and deprivation) for stroke in relation to eight mental health morbidities. The unadjusted and adjusted ORs, along with the crude prevalence of all mental health morbidities in the stroke and stroke-free groups, are shown in supplementary material (see Additional file 5). In all, six of the eight mental health morbidities examined were significantly more common in the stroke group, and following adjustments, all eight mental health morbidities were significantly more common. These included drug and medication use problems (adjusted OR 2.34; 95% CI 2.25 to 2.43), depression (adjusted OR 2.09; 95% CI 2.03 to 2.15), alcohol problems (adjusted OR 2.05; 95% CI 1.96 to 2.15) and anxiety and stress (adjusted OR 1.61; 95% CI 1.55 to 1.66).

### Regular prescriptions

As anticipated, the number of regular prescriptions was significantly correlated with number of morbidities in the stroke (Spearman’s ρ = 0.58 *P* < 0.001) and control (Spearman’s ρ = 0.75 *P* < 0.001) groups. Table 2 shows the number of repeat prescriptions in the stroke and control groups, and the ORs. Those with stroke were more likely than the controls to be on a repeat prescription (adjusted OR 4.53; 95% CI 4.33 to 4.74). In the stroke group, 12.6% had eleven or more repeat prescriptions compared with only 1.5% of the control group (OR adjusted for age, sex, deprivation and morbidity count 15.84; 95% CI 14.86 to 16.88).

**Table 2:** **Stroke status and number of repeat medications (N = 1,424,378)**

	**Stroke N (%)**	**No stroke N (%)**	**Unadjusted OR (95% ** **CI)** ^**a**^	**Age, gender and deprivation adjusted OR (95%** **CI)** ^**a**^	**Age, gender, deprivation and morbidity count adjusted OR (95%** **CI)** ^**a**^
	**35690 (100)**	**1388688 (100)**			
Number of medications					
None	2447 (6.9%)	863688 (62.2%)	1	1	1
One-two	3038 (8.5%)	240721 (17.3%)	4.45 (4.22 to 4.70)	2.38 (2.26 to 2.52)	2.29 (2.17 to 2.42)
Three-four	6566 (18.4%)	122518 (8.8%)	18.92 (18.05 to 19.82)	6.25 (5.95 to 6.57)	5.78 (5.49 to 6.08)
Five-six	8185 (22.9%)	75512 (5.4%)	38.26 (36.55 to 40.05)	10.50 (9.99 to 11.03)	9.36 (8.89 to 9.86)
Seven-eight	6721 (18.8%)	43344 (3.1%)	54.73 (52.20 to 57.38)	13.90 (13.20 to 14.63)	11.94 (11.29 to 12.62)
Nine-ten	4219 (11.8%)	22536 (1.6%)	66.08 (62.76 to 69.57)	16.22 (15.34 to17.15)	13.44 (12.65 to 14.29)
Eleven or more	4514(12.6%)	20369 (1.5%)	78.22 (74.32 to 82.32)	20.13 (19.05 to 21.27)	15.84 (14.86 to 16.88)

^a^all p < 0.001.

## Discussion

### Summary of findings and implications

Analyses of a large, nationally representative sample of people in Scotland, a country with universal healthcare, showed that multimorbidity and polypharmacy were more common in people with a diagnosis of stroke. These findings are consistent with our knowledge that those with stroke are an elderly population with considerable cardiovascular disease risk [44], for whom effective treatments are increasingly available to alleviate symptoms and address underlying causal factors [45]. Diagnoses of most chronic conditions were more common in the stroke group, and this remained the case after adjustment for age, sex and deprivation. In our preliminary analyses (see Additional file 2), both age and deprivation were associated with stroke in the expected directions. This gives us confidence in the novel results presented herein.

Polypharmacy represents only one aspect of treatment burden, but is directly measurable, and may be a proxy measure of wider aspects of burden [17,18]. Multimorbidity is likely to increase treatment burden in several ways. First, as this study and others have shown, the number of medications increases with number of conditions [20,21]. Second, treatments may interact, leading to side effects [5,7,46] and this has the potential to further increase the volume of work; for example, as new treatments are given to compensate for interactions [47]. Third, multimorbidity is likely to increase healthcare contacts and affect the capacity of the individual to follow therapeutic regimens [48]; for example, those with stroke and comorbid arthritis may find physiotherapy sessions more challenging [49,50]. Fourth, multimorbid patients who become overburdened, for example by complex medication regimens, may be less likely to adhere to therapies, leading to poor disease control and a further escalation of treatments by health professionals, further increasing treatment burden [3,9,51]. While many pharmacological therapies may be beneficial for those with stroke, a key question is whether people with stroke have made informed decisions regarding whether or not to take so many medications, given their modest benefits. Although perceived treatment burden and capacity to cope with any given treatment burden will vary, we would recommend that patients with stroke are made aware of the relative benefits of their drugs, and are empowered to make their own decision whether to take them.

Acknowledging and addressing treatment burden in stroke, particularly for those with multimorbidity, may improve the patient experience, adherence to therapies, and health outcomes [48]. Minimising unnecessary treatments, improving co-coordination of services and making care more patient-centred [23] are likely to lessen treatment burden, but will necessitate changes from policy level down to the individual consultation [3,48,52,53]. Most stroke management guidelines fail to mention multimorbidity, or merely acknowledge the more common comorbidities briefly with a lack of practical advice for clinicians [45,54-57]. We found only one stroke guideline that acknowledged the issue of polypharmacy, and again, detailed practical help was lacking [56]. This issue has been gaining prominence [58,59]. Guidelines should be redesigned to take account of comorbidity and treatment burden; for example, by providing guidance on potential interactions from drug combinations commonly prescribed for those with stroke and multimorbidity and how to deal with the possible side effects or interactions that may arise [47]. In the current study, 21.9% of people with stroke had a painful condition, 20.7% had depression and 13.0% had atrial fibrillation, increasing the risk of being prescribed non-steroidal anti-infammatory drugs (NSAIDs), anti-depressants, anti-platelet therapies and anti-coagulants concomitantly, which increases risk of adverse events, such as bleeding. Care pathways should be structured around the patient themselves, rather than the individual conditions, using a more generalist approach that considers issues such as multimorbidity as well as the individual’s support network and financial resources [9,60,61].

### Strengths and limitations

This analysis was undertaken using data from a large, nationally representative, primary care sample, and as far as we are aware, this is the first study on such a scale to examine multimorbidity and polypharmacy in stroke. This sample is representative of the Scottish population [37]; however, it may not reflect experience in other countries and healthcare systems. The prevalence of stroke in this sample was similar to that shown in other studies [44,62], further validating the data; however, the data were collected for clinical rather than research purposes. No standard methods for measuring multimorbidity or polypharmacy exist, therefore a pragmatic approach was taken. We examined thirty nine long-term conditions, which is substantially more than in previous studies. The rationale for including the conditions examined and the rules for identifying the presence of each were described in detail by the team who previously collated the data [1]. In addition, any medications bought over the counter or given from secondary care were not included. However, at the time of the analysis, prescriptions to people over sixty five years of age and to many people with chronic conditions were all free, with others being able to cap their out-of-pocket costs, thus suggesting a financial incentive to obtain medication via the primary care practice.

As this is a cross-sectional study, the data we have were taken from one particular point in time, and therefore no conclusions about temporality or causation can be made. The measure of comorbidity was unweighted, as the aim was to be descriptive rather than to assess outcomes. This was deemed to be the most appropriate method, and is similar to that used by others investigating the prevalence of multimorbidity [1], but could be viewed as a limitation, especially as there may be a qualitative difference between the effects on perceived treatment burden of long-term conditions that produce regular symptoms (for example, heart failure) and those that are asymptomatic (for example, hypertension). We have no information about stroke severity, which is also a potential limitation. It should also be noted that due to the nature of the study, multiple analyses were carried out. Thus, the large numbers of cases and controls assessed in this study may have identified some associations that were statistically significant but not necessarily clinically significant; for example, for conditions such as cancer, glaucoma and asthma, which had ORs between 1.08 and 1.10 but were statistically significant with *P* < 0.001.

Lastly, to explore treatment burden in stroke, this study examined multimorbidity and polypharmacy, however there are many more aspects of treatment burden still to be examined, such as clinic visits, continuity, coordination of care, and financial burden of therapies. The development of a patient-reported measure would enable a more detailed examination of treatment burden in stroke from the patient perspective.

## Conclusion

In this study, we found that multimorbidity and polypharmacy were strikingly more common in those with stroke than those without. Polypharmacy can be thought of as a direct measure of one aspect of treatment burden, and we would suggest that people with stroke should be made aware of the relative benefits of their drugs so they can make informed decisions about therapeutic regimens. Both polypharmacy and multimorbidity are likely to be proxy markers for other aspects of treatment burden, as patients face the demands of managing multiple medications and conditions simultaneously. Clinical guidelines for stroke need to place greater emphasis on the management of multimorbidity, and further investigation of treatment burden in stroke is required to inform redesign of health services to improve patient outcomes.

## Additional files

Additional file 1:
**Definitions of morbidities assessed.**
Additional file 2:
**Stroke status in relation to demographic characteristics (n = 1,424,378).**
Additional file 3:
**Stroke status and number of morbidities and repeat medications for two age groups.**
Additional file 4:
**Stroke status and prevalence of physical morbidities (n = 1,424,378).**
Additional file 5:
**Stroke status and prevalence of mental health morbidities (n = 1,424,378).**

## Abbreviations

AF: Atrial fibrillation
CHD: Coronary heart disease
NHS: National Health Service
PCCIU: Primary Care Clinical Informatics Unit
QOF: Quality and Outcomes Framework
