# Supplementary material for: Stroke, multimorbidity and polypharmacy in a nationally representative sample of 1,424,378 patients in Scotland: implications for treatment burden
Source: BMC Med. 2014 Oct 3;12:151. doi: 10.1186/s12916-014-0151-0 (PMC4220053; doi:10.1186/s12916-014-0151-0)
Supplement: Additional file 1: — Definitions of morbidities assessed. [file 12916_2014_151_MOESM1_ESM.docx]

**Additional File 1.** **Definitions of morbidities assessed**

| **Condition** | **Variable Definition** |
| --- | --- |
| Coronary heart disease | Read code ever recorded |
| Chronic kidney disease | Read code ever recorded |
| Asthma (active) | Read code ever recorded AND any prescription in last year |
| Atrial fibrillation | Read code ever recorded |
| Epilepsy | Read code ever recorded AND epilepsy prescription in last year |
| New cancer in the last 5 years | Read code first recorded in last 5 years (Relevant Read Code recorded) |
| Thyrotoxicosis/Thyroid disorders (includes hypothyroidism) | Read code ever recorded (Relevant Read Code recorded) |
| Diabetes | Read code ever recorded |
| Parkinson’s disease | Read code ever recorded (Relevant Read Code recorded) |
| Multiple sclerosis | Read code ever recorded (Relevant Read Code recorded) |
| Visual impairment | Read code ever recorded (Relevant Read Code recorded) |
| Glaucoma | Read code ever recorded (Relevant Read Code recorded) |
| Hearing loss | Read code ever recorded (Relevant Read Code recorded) |
| Hypertension | Read code ever recorded (Relevant Read Code recorded) |
| Heart failure | Read code ever recorded |
| Peripheral vascular diseases | Read code ever recorded (Relevant Read Code recorded) |
| Chronic sinusitis | Read code ever recorded (Relevant Read Code recorded) |
| Bronchitis, emphysema & other chronic obstructive pulmonary diseases | Read code ever recorded (Relevant Read Code recorded) |
| Bronchiectasis | Read code ever recorded (Relevant Read Code recorded) |
| Crohn’s disease & ulcerative colitis | Read code ever recorded (Relevant Read Code recorded) |
| Diverticular disease of intestine | Read code ever recorded (Relevant Read Code recorded) |
| Rheumatoid arthritis, other inflammatory polyarthropathies & systematic connective tissue disorders | Read code ever recorded (Relevant Read Code recorded) |
| Hyperplasia of prostate & prostate disorders | Read code ever recorded (Relevant Read Code recorded) |
| Psoriasis or eczema | Read code ever recorded (M11% & M12%) AND ≥ 4 prescription in last year (BNF 13.4, excluding hydrocortisone, & BNF 13.5) |
| Viral Hepatitis | Read code ever recorded (Relevant Read Code recorded) |
| Irritable bowel syndrome | Read code ever recorded (Relevant Read Code recorded) OR ≥ 4 antispasmodic prescription in last year (POM only, exclude kolanticon, alverine citrate & peppermint oil) |
| Cirrhosis/chronic liver disease/alcoholic liver disease | Read code ever recorded (Relevant Read Code recorded) |
| Migraine | ≥ 4 anti-migraine prescriptions in last year (BNF 040704%, POM only exclude migraleve) |
| Dyspepsia | ≥ 4 prescriptions in last year BNF 0103% excluding antacids AND NOT ≥4 NSAIDS OR ≥4 aspirin/clopidogrel |
| Constipation | ≥4 prescriptions in last year, BNF 0106% |
| Pain | ≥4 specified analgesic prescriptions in last year (opioids/>8mg co-codamol/NSAIDS) OR ≥4 specified anti-epileptics in the absence of an epilepsy Read code in last year (gabapentin, pregabalin and carbamazepine) |
| Anorexia or bulimia | Read code ever recorded |
| Drugs misuse (Other psychoactive substance misuse) | Read code ever recorded |
| Anxiety & other neurotic, stress related & somatoform disorders | Read code in last 12 months OR ≥ 4 anxiolytic/hypnotic prescriptions in last 12 months OR ≥ 4 10/25mg amitriptyline in last 12 months & do not meet the criteria for ‘Pain’ |
| Depression | Read code recorded in last 12 months OR ≥4 anti-depressant prescriptions (excluding low dose tricyclics) in last 12 months |
| Alcohol misuse | Read code ever recoded |
| Schizophrenia (and related non-organic psychosis) or bipolar disorder | Read code ever recorded/recorded in last 12 months (code dependent) OR Lithium  prescribed in last 168 days |
| Dementia | Read code ever recorded |
| Learning disability | Read code ever recorded |
