# Supplementary material for: Stroke, multimorbidity and polypharmacy in a nationally representative sample of 1,424,378 patients in Scotland: implications for treatment burden
Source: BMC Med. 2014 Oct 3;12:151. doi: 10.1186/s12916-014-0151-0 (PMC4220053; doi:10.1186/s12916-014-0151-0)
Supplement: Additional file 2: — Stroke status in relation to demographic characteristics (n = 1,424,378). [file 12916_2014_151_MOESM2_ESM.docx]

**Additional File 2. Stroke status in relation to demographic characteristics (N=1,424,378)**

|  | **Stroke**  **N (%)**  **35690 (100)** | **No stroke**  **N (%)**  **1388688(100)** | **Unadjusted OR**  **(95% CI) ^a^** | **Adjusted OR (95% CI) ^a,b^** |
| --- | --- | --- | --- | --- |
| **Men** | 18071 (50.6) | 681358 (49.1) | 1.07 (1.04 to 1.09)^c^ | 1.49 (1.46 to 1.53)^c^ |
| **Age group**  **18-24**  **25-34**  **35-44**  **45-54**  **55-64**  **65-74**  **75+** | 50 (0.1)  157 (0.4)  592 (1.7)  2019 (5.7)  5650 (15.8)  9847 (27.6)  17375 (48.7) | 151643 (10.9)  229239 (16.5)  278401 (20.0)  251775 (18.1)  213683 (15.4)  145433 (10.5)  118514 (8.5) | 1  2.08 (1.51 to 2.86)  6.45 (4.83 to 8.61)  24.32 (18.37 to 32.20)  80.19 (60.70 to 105.94)  205.35 (155.51 to 271.16)  444.64 (336.83 to 586.95) | 1  2.08 (1.52 to 2.86)  6.53 (4.89 to 8.71)  24.80 (18.73 to 32.83)  82.58 (62.51 to 109.10)  212.61 (161.01 to 280.74)  476.65 (361.07 to 629.23) |
| **Deprivation quintile**  **1 affluent**  **2**  **3**  **4**  **5 deprived** | 5993 (16.8)  7724 (21.6)  8251 (23.1)  7008 (19.6)  6714 (18.8) | 266036 (19.2)  296430 (21.3)  313996 (22.6)  264376 (19.0)  247850 (17.8) | 1  1.16 (1.12 to 1.20)  1.17 (1.13 to 1.21)  1.18 (1.14 to 1.22)  1.20 (1.16 to 1.25) | 1  1.09 (1.05 to 1.12)  1.20 (1.16 to 1.24)  1.28 (1.24 to 1.33)  1.42 (1.37 to 1.47) |
| ^a^ all p values <0.001  ^b^ adjustments were made for all other demographic variables e.g. age and deprivation when examining gender.  ^c^ reference category is women | | | | |
