# Supplementary material for: Stroke, multimorbidity and polypharmacy in a nationally representative sample of 1,424,378 patients in Scotland: implications for treatment burden
Source: BMC Med. 2014 Oct 3;12:151. doi: 10.1186/s12916-014-0151-0 (PMC4220053; doi:10.1186/s12916-014-0151-0)
Supplement: Additional file 3: — Stroke status and number of morbidities and repeat medications for two age groups. [file 12916_2014_151_MOESM3_ESM.docx]

**Additional File 3. Stroke status and number of morbidities and repeat medications for two age groups**

|  | **Stroke N (%)**  **35690 (100)** | **No stroke (%)**  **1388688 (100)** | **Gender and deprivation adjusted OR (95% CI) ^a^** |
| --- | --- | --- | --- |
| **Total number of morbidities^b^ (Age group 35-44)**  **None**  **One-three**  **Four-six**  **Seven or more** | 162 (27.4)  332 (56.1)  84 (14.2)  14 (2.4) | 179346 (64.4)  92652 (33.3)  6069 (2.2)  334 (0.1) | 1  3.93 (3.25 to 4.75)  14.77 (11.29 to 19.32)  45.19 (25.83 to 79.04) |
| **Number of morbidities^b^ (Age group 75 plus)**  **None**  **One-three**  **Four-six**  **Seven or more** | 435 (2.5)  7620 (43.9)  7009 (40.3)  2311 (13.3) | 11846 (10.0)  64934 (54.8)  34197 (28.9)  7537 (6.4) | 1  3.23 (2.92 to 3.56)  5.69 (5.15 to 6.28)  8.56 (7.70 to 9.53) |
| **Number of medications (Age 35-44)**  **None**  **One-two**  **Three-four**  **Five-six**  **Seven-eight**  **Nine-ten**  **Eleven or more** | 180 (30.4)  135 (22.8)  99 (16.7)  65 (11.0)  46 (7.8)  37 (6.3)  30 (5.1) | 217,943 (78.3)  42,772 (15.4)  10,688 (3.8)  3,912 (1.4)  1,713 (0.6)  735 (0.3)  638 (0.2) | 1  3.86 (3.06 to 4.83)  11.12 (8.68 to 14.25)  19.83 (14.89 to 26.42)  32.10 (23.10 to 44.60)  59.91 (41.66 to 86.16)  56.14 (37.77 to 83.43) |
| **Number of medications (Age 75 plus)**  **None**  **One-two**  **Three-four**  **Five-six**  **Seven-eight**  **Nine-ten**  **Eleven or more** | 970 (5.6)  1,017 (5.9)  2,897 (16.7)  4,147 (23.9)  3,690 (21.2)  2,376 (13.7)  2,278 (13.1) | 16,153 (13.6)  20,805 (17.6)  26,558 (22.4)  23,290 (19.7)  15,616 (13.2)  8,760 (7.4)  7,332 (6.2) | 1  0.82 (0.75 to 0.90)  1.85 (1.72 to 2.00)  3.04 (2.83 to 3.27)  4.05 (3.76 to 4.36)  4.68 (4.32 to 5.07)  5.38 (4.97 to 5.83) |
| ^a^ all p<0.001  ^b^ excluding stroke | | | |
