# Supplementary material for: Stroke, multimorbidity and polypharmacy in a nationally representative sample of 1,424,378 patients in Scotland: implications for treatment burden
Source: BMC Med. 2014 Oct 3;12:151. doi: 10.1186/s12916-014-0151-0 (PMC4220053; doi:10.1186/s12916-014-0151-0)
Supplement: Additional file 4: — Stroke status and prevalence of physical morbidities (n = 1,424,378). [file 12916_2014_151_MOESM4_ESM.docx]

**Additional File 4. Stroke status and prevalence of physical morbidities (N=1,424,378)**

|  | **Stroke N (%)**  **35690 (100)** | **No stroke N (%)**  **1388688 (100)** | **Unadjusted OR (95% CI)^a,b^** | **Age, gender and deprivation adjusted OR (95% CI) ^a,b^** |
| --- | --- | --- | --- | --- |
| **Epilepsy** | 1186 (3.3) | 11198 (0.8) | 4.23 (3.98 to 4.49) | 4.43 (4.14 to 4.74) |
| **Hypertension** | 21742 (60.9) | 212572 (15.3) | 8.62 (8.44 to 8.81) | 2.67 (2.61 to 2.73) |
| **Peripheral vascular diseases** | 3286 (9.2) | 19954 (1.4) | 6.96 (6.69 to 7.23) | 2.47 (2.37 to 2.58) |
| **Atrial fibrillation** | 4652 (13.0) | 19324 (1.4) | 10.62 (10.27 to 10.99) | 2.44 (2.36 to 2.53) |
| **Coronary heart disease** | 10518 (29.5) | 70949 (5.1) | 7.76 (7.58 to 7.95) | 2.06 ( 2.01 to 2.11) |
| **Diabetes** | 6697 (18.8) | 68134 (4.9) | 4.48 (4.36 to 4.60) | 1.96 (1.90 to 2.02) |
| **Migraine** | 273 (0.8) | 8978 (0.6) | 1.19 (1.05 to 1.34)  p=0.006 | 1.87 (1.65 to 2.12) |
| **Chronic liver disease** | 159 (0.4) | 2455 (0.2) | 2.53 (2.15 to 2.97) | 1.86 (1.57 to 2.19) |
| **Chronic kidney disease** | 5100 (14.3) | 28466 (2.0) | 7.97 (7.72 to 8.23) | 1.85 (1.78 to 1.91) |
| **Heart failure** | 2977 (8.3) | 15922 (1.1) | 7.85 (7.53 to 8.17) | 1.82 (1.74 to 1.90) |
| **Constipation** | 4934 (13.8) | 31482 (2.3) | 6.92 (6.70 to 7.14) | 1.74 (1.68 to 1.80) |
| **Viral Hepatitis** | 19 (0.1) | 1156 (0.1) | 0.64 (0.41 to 1.01)  p=0.053 | 1.72 (1.08 to 2.74)  p=0.023 |
| **Blindness & low vision** | 1124 (3.1) | 7254 (0.5) | 6.19 (5.81 to 6.60) | 1.59 (1.48 to 1.70) |
| **Painful condition** | 7810 (21.9) | 118321 (8.5) | 3.01 (2.93 to 3.09) | 1.51 (1.47 to 1.55) |
| **Chronic obstructive pulmonary disease** | 4235 (11.9) | 48872 (3.5) | 3.69 (3.57 to 3.82) | 1.44 (1.39 to 1.50) |
| **Inflammatory arthritis, connective tissue disorders and gout** | 4198 (11.8) | 53810 (3.9) | 3.31 (3.20 to 3.42) | 1.40 (1.35 to 1.45) |
| **Diverticular disease of intestine** | 3472 (9.7) | 30341 (2.2) | 4.83 (4.65 to 5.01) | 1.35 (1.29 to 1.40) |
| **Thyroid disorders** | 4244 (11.9) | 67699 (4.9) | 2.63 (2.55 to 2.72) | 1.34 (1.29 to 1.39) |
| **Psoriasis or eczema** | 446 (1.2) | 9923 (0.7) | 1.76 (1.60 to 1.93) | 1.32 (1.19 to 1.46) |
| **Parkinson’s disease** | 361 (1.0) | 2380 (0.2) | 5.95 (5.33 to 6.65) | 1.30 (1.16 to 1.46) |
| **Inflammatory bowel disease** | 382 (1.1) | 9369 (0.7) | 1.59 (1.44 to 1.77) | 1.27 (1.14 to 1.41) |
| **Irritable bowel syndrome** | 1539 (4.3) | 50598 (3.6) | 1.19 (1.13 to 1.26) | 1.23 (1.17 to 1.30) |
| **Hearing loss** | 3968 (11.1) | 50766 (3.7) | 3.30 (3.19 to 3.41) | 1.17 (1.13 to 1.22) |
| **Multiple sclerosis** | 92 (0.3) | 3755 (0.3) | 0.95 (0.78 to 1.17)  p=0.650 | 1.12 (0.90 to 1.38)  p=0.309 |
| **Prostate disorders** | 1424 (4.0) | 13809 (1.0) | 4.14 (3.91 to 4.37) | 1.11 (1.05 to 1.18)  p=0.001 |
| **Asthma** | 2285 (6.4) | 82220 (5.9) | 1.09 (1.04 to 1.14) | 1.10 (1.05 to 1.15) |
| **Glaucoma** | 1544 (4.3) | 14375 (1.0) | 4.32 (4.10 to 4.56) | 1.08 (1.02 to 1.14) |
| **Cancer** | 2937 (8.2) | 40727 (2.9) | 2.97 (2.85 to 3.09) | 1.08 (1.04 to 1.13) |
| **Bronchiectasis** | 173 (0.5) | 2641 (0.2) | 2.56 (2.19 to 2.98) | 1.06 (0.91 to 1.25)  p=0.454 |
| **Chronic sinusitis** | 248 (0.7) | 8917 (0.6) | 1.08 (0.95 to 1.23)  p=0.219 | 1.02 (0.89 to 1.16)  p=0.820 |
| **Dyspepsia** | 2571 (7.2) | 76633 (5.5) | 1.33 (1.28 to 1.38) | 0.63 (0.60 to 0.66) |
| ^a^ reference category is absence of each condition  ^b^ all p values <0.001 unless otherwise stated | | | | |
