# Supplementary material for: Stroke, multimorbidity and polypharmacy in a nationally representative sample of 1,424,378 patients in Scotland: implications for treatment burden
Source: BMC Med. 2014 Oct 3;12:151. doi: 10.1186/s12916-014-0151-0 (PMC4220053; doi:10.1186/s12916-014-0151-0)
Supplement: Additional file 5: — Stroke status and prevalence of mental health morbidities (n = 1,424,378). [file 12916_2014_151_MOESM5_ESM.docx]

**Additional File 5. Stroke status and prevalence of mental health morbidities (N=1,424,378)**

|  | **Stroke N (%)**  **35690 (100)** | **Non stroke N (%)**  **1388688 (100)** | **Unadjusted OR (95% CI) ^a,b^** | **Age, gender and deprivation adjusted OR (95% CI) ^a,b^** |
| --- | --- | --- | --- | --- |
| **Drug and medication use problems** | 3831 (10.7) | 38629 (2.8) | 4.20 (4.06 to 4.35) | 2.34 (2.25 to 2.43) |
| **Depression** | 7394 (20.7) | 136549 (9.8) | 2.40 (2.33 to 2.46) | 2.09 (2.03 to 2.15) |
| **Alcohol problems** | 2062 (5.8) | 40301 (2.9) | 2.05 (1.96 to 2.15) | 2.05 (1.96 to 2.15) |
| **Anxiety & stress** | 4026 (11.3) | 51700 (3.7) | 3.29 (3.18 to 3.40) | 1.61 (1.55 to 1.66) |
| **Learning disability** | 115 (0.3) | 4899 (0.4) | 0.91 (0.76 to 1.10)  p=0.336 | 1.50 (1.24 to 1.82) |
| **Anorexia or bulimia** | 118 (0.3) | 5188 (0.4) | 0.89 (0.74 to 1.06)  p=0.189 | 1.50 (1.24 to 1.83) |
| **Dementia** | 2075 (5.8) | 9621 (0.7) | 8.85 (8.43 to 9.29) | 1.44 (1.37 to 1.52) |
| **Schizophrenia and bipolar disorder** | 452 (1.3) | 12041 (0.9) | 1.47 (1.33 to 1.61) | 1.32 (1.20 to 1.46) |
| ^a^ reference category is absence of each condition  ^b^ all p values <0.001 unless otherwise stated | | | | |
